# Microbial Biomarkers for the Prevention and Diagnosis of Alcoholic Liver Disease

**DOI:** 10.3390/microorganisms14020449

**Published:** 2026-02-12

**Authors:** Goo Hyun Kwon, Hyunjoon Park, Hyeong Seop Kim, Ki Kwang Oh, Jung A Eom, Kyeong Jin Lee, Min Ju Kim, Minsoo Kim, Jeong Su Kim, Sang Hak Han, Young Lim Ham, Ki Tae Suk

**Affiliations:** 1 Institute for Liver and Digestive Diseases, Hallym University, Chuncheon 24252, Republic of Korea; ninetjd@naver.com (G.H.K.); hyunjoons.park@gmail.com (H.P.); kimhs2425@gmail.com (H.S.K.); nivirna07@kangwon.ac.kr (K.K.O.); eomjunga32@naver.com (J.A.E.); rudwls1134@naver.com (K.J.L.); kminju1119@naver.com (M.J.K.); ms120567@hallym.ac.kr (M.K.); jungsu052518@naver.com (J.S.K.); 2 Department of Pathology, Hallym University Chuncheon Sacred Heart Hospital, Chuncheon 24253, Republic of Korea; drhsh74@hallym.or.kr; 3 Department of Nursing, Daewon University College, Jecheon 27137, Republic of Korea; ylham@daewon.ac.kr

**Keywords:** alcohol-associated liver disease, inflammation, gut microbiota, biomarker

## Abstract

Alterations in gut microbiota are closely associated with alcohol-associated liver disease (ALD) progression. We aimed to identify ALD-related bacterial strains with therapeutic or diagnostic potential. Human fecal samples were analyzed to screen candidate microbes, and an ALD mouse model was used to evaluate their effects. We also assessed bacterial DNA levels in blood to explore diagnostic utility. *Lactobacillus helveticus* and *L. lactis* treatment improved gut dysbiosis and reduced hepatic inflammation and endotoxemia. In contrast, *Veillonella dispar*, which is significantly enriched in ALD patients, had no beneficial effects in vivo. Instead, *V. dispar* abundance in blood distinguished ALD patients from controls with an area under the ROC curve of 0.815. These findings suggest that *L. helveticus* and *L. lactis* may be effective probiotics for ALD, while *V. dispar* may serve as a non-invasive diagnostic biomarker. Targeting microbiota may offer a new approach for ALD prevention and diagnosis.

## 1. Introduction

Harmful use of alcohol is a leading risk factor for disease and disability worldwide, causing 2.6 million deaths annually and accounting for 4.7% of all deaths [1]. Alcohol-associated liver disease (ALD) encompasses a spectrum of conditions ranging from asymptomatic hepatic steatosis to hepatitis, fibrosis, cirrhosis, and hepatocellular carcinoma [2]. Infection and cell death are the two most common drivers of inflammation, and increasing evidence implicates inflammation as a central mechanism in ALD progression [3,4]. Despite the rising global prevalence of ALD [1], early diagnosis remains a significant clinical challenge. Current gold-standard diagnostic methods, such as liver biopsy, are invasive, costly, and prone to sampling errors. Consequently, there is an urgent unmet need for the development of non-invasive biodiagnostic markers that can accurately assess disease severity and progression [2]. The translocation of pathogen-associated molecular patterns (PAMPs), such as bacterial endotoxins, from the gut into the circulation represents a key inflammatory signal, activating hepatic immune responses and promoting liver injury [5]. Accumulating evidence indicates that treatment with symbiotic bacterial species, including *Lactobacillus* spp., can ameliorate alcohol-induced steatosis and gut microbiota dysbiosis [6,7,8]. Probiotics are defined as live microorganisms that, when administered in adequate amounts, confer health benefits to the host. Probiotics and their metabolites can directly or indirectly modulate host metabolic processes and immune function [9]. Administration of *Lactobacillus rhamnosus GG* for two weeks reduced endotoxemia and hepatic steatosis in C57BL/6N mice [10]. As highlighted in a recent systematic review, in a clinical trial, *Bifidobacterium bifidum* and *L. plantarum* supplementation significantly decreased serum aspartate transaminase (AST) and alanine transaminase (ALT) levels in patients with ALD within five days of treatment [11]. These findings suggest that the gut microbiota plays an important role in regulating the pathogenesis of ALD. Recently, increasing attention has been directed toward the role of the microbiome in the development of ALD. Through 16S rRNA gene sequencing, human microbiome profiling studies have advanced our understanding of the gut–liver axis and highlighted the therapeutic potential of microbiota-targeted interventions in ALD [12]. Furthermore, distinct blood microbiome signatures linked to metabolic dysfunction have been observed both in heavy drinkers without advanced liver disease and in patients with alcoholic hepatitis [13].

In this study, we adopted a dual-track approach to fill this gap. We aimed to determine whether specific probiotics (*L. helveticus* and *L. lactis*) could mechanistically attenuate ALD-induced inflammation and, concurrently, whether specific microbiome-derived signals (*V. dispar*) could serve as novel, non-invasive diagnostic biomarkers. This integrated strategy offers a comprehensive perspective on both the prevention and diagnosis of ALD.

## 2. Materials and Methods

### 2.1. Patient Population

A prospective cohort study was conducted between April 2017 and March 2020 (ClinicalTrials.gov: NCT04339725) at the hepatology department of Hallym University. A total of 224 subjects were enrolled and categorized into four groups: healthy controls (*n* = 56), alcohol consumption without liver failure (*n* = 46), alcoholic hepatitis (AH; *n* = 57), and alcoholic liver cirrhosis (ALC; *n* = 65). Patients received standard treatment for their respective conditions, independent of study participation (Supplementary Figure S1). Participants who had used medications known to alter gut microbiota (e.g., antibiotics, proton pimp inhibitors, probiotics, prebiotics, or chemotherapeutic agents) at the time of enrollment were excluded. ALD was diagnosed based on the results of alcohol history, liver biopsy, blood chemistry, and imaging studies (ultrasound or computed tomography scan). The ALD group was subgrouped into AH and ALC. AH patients were defined as those with abnormal liver enzymes (AST ≥ 50 IU/L, AST/ALT > 1.5, and AST and ALT < 400 IU/L) and excessive alcohol consumption (male > 60 g/day and female > 40 g/day) for a duration of at least 5 years, with their last alcohol drink within 8 weeks of jaundice onset (bilirubin > 3 mg/dL). The control group was recruited from a medical check-up center. Exclusion criteria included a history of viral hepatitis, autoimmune hepatitis, pancreatitis, hemochromatosis, Wilson’s disease, cancer, or drug-induced liver injury.

The study protocol adhered to the ethical guidelines of the 1975 Helsinki Declaration and was approved by the institutional review board of Hallym University (2016-134). Written informed consent was obtained from all participants.

Baseline assessments included complete blood counts, liver function tests, and viral markers. Patients with AH or ALC additionally underwent abdominal ultrasound or computed tomography. Serum biochemical parameters included AST, ALT, creatinine, cholesterol, γ-glutamyl transpeptidase (γGTP), triglycerides (TGs), and high-density lipoprotein cholesterol (HDL-C). Stool samples were collected from all participants: controls provided samples at the time of medical examination, while patients submitted samples upon hospital admission. All stool specimens were stored at −80 °C until analysis. Clinical data were integrated with metagenomics profiles for subsequent analysis.

### 2.2. Stool Sample Collection and Analysis

Genomic DNA was extracted from stool samples using the QIAamp DNA Stool Mini Kit (Qiagen, Hilden, Germany). Libraries were prepared with the NEBNext Ultra II FS DNA Library Prep Kit for Illumina (New England BioLabs, Ipswich, MA, USA) following the manufacturer’s instructions. Library concentrations were measured using the Qubit dsDNA HS Assay Kit (Thermo Fisher Scientific, Waltham, MA, USA) and validated by qPCR with the KAPA SYBR FAST qPCR Master Mix Kit (Kapa Biosystems, Wilmington, MA, USA). Library quality was assessed using the Agilent 2100 Bioanalyzer (Agilent Technologies, Santa Clara, CA, USA) with a DNA 12000 chip. Sequencing was performed on the Illumina NovaSeq 6000 platform (PE 150 bp) at ChunLab, Inc. (Seoul, Republic of Korea). Additional sequencing runs were carried out on the Illumina MiSeq platform with reagent kit V3 in PE 250 bp mode. Microbiome taxonomic profiling was conducted using the EZBioCloud platform (ChunLab, Seoul, Republic of Korea; database version PKSSU4.0). Comparative analyses were performed with the MTP analyzer module of EZBioCloud. Operational taxonomic units (OTUs) were clustered using UCLUST and CD-HIT with a 97% similarity cutoff. Alpha-diversity indices (Good’s coverage, rarefaction, ACE, Chao1, Jackknife, Shannon, Simpson, and NPShannon) were calculated, while beta-diversity was assessed using the unweighted pair group method with arithmetic mean (UPGMA) clustering and principal coordinate analysis (PCoA).

### 2.3. Bacteria Culture

The *Lactobacillus* strains used in the preliminary study were isolated from various sources, including sour milk, cheese, the feces of healthy Korean adults, and newborn feces. *L. helveticus* and *L. lactis*, originally isolated from sour milk, were cultured in de Man, Rogosa, and Sharpe (MRS) broth (BD/Difco) at 37 °C under anaerobic conditions for 24 h. Stock cultures were prepared by mixing the broth with 20% skim milk (*v*/*v*) and storing them at −80 °C. Seed cultures were prepared in MRS broth at 37 °C for 24 h, followed by inoculation into optimized medium in a fermenter (MARADO-05D-PS, Bio Control & Science, Daejeon, Republic of Korea). Fermentation was carried out at 37 °C for 18–20 h with constant agitation (120 rpm) and pH was maintained at 5.5–6.0 by automatic addition of 25% NaOH solution. Cells were harvested by centrifugation at 6000 rpm for 10 min (Supra R12, Hanil Scientific, Gimpo, Republic of Korea) and lyophilized (Lab-Mast 10, Cooling & Heating System, Busan, Republic of Korea) according to the manufacturer’s instructions. Colony-forming units (CFU) per gram of probiotic powder were quantified by serial dilution. For *Veillonella dispar*, cultures were grown on reinforced clostridial medium (RCM; Difco) supplemented with 1.5% agar and sodium lactate (7.5 g/L; Samchun Pure Chemical, Pyeongtaek, Republic of Korea, Cat. No. S2236). Plates were incubated under anaerobic conditions until reaching a cell density of ~10^9^ CFU. Bacterial suspensions were prepared in 0.1 M PBS and adjusted to 10^9^ CFU/mL prior to use.

### 2.4. Animal Model of ALD

All animal experiments were conducted in accordance with the National Institutes of Health Guidelines for the Care and Use of Laboratory Animals and were approved by the Institutional Animal Care and Use Committee of the College of Medicine, Hallym University (2018-04). Six-week-old specific-pathogen-free (SPF) male C57BL/6J mice were purchased from Dooyeol Biotech (Dooyeol Biotech, Seoul, Republic of Korea). Mice were individually housed in stainless steel microisolator cages under controlled conditions (22 ± 2 °C, 12 h light/dark cycle) with ad libitum access to food and water. All animals were acclimatized for one week on a standard chow diet before the start of the experiment. Thereafter, mice were randomly assigned to receive either an ethanol-containing Lieber–DeCarli liquid diet (EtOH-fed) or an isocaloric control liquid diet (control) for 8 weeks, as previously described. After a one-week acclimatization period on a control diet, ethanol feeding was initiated at 23.91 g/kg/day and incrementally increased every two days to 24.64 g/kg/day by days 9–14. At this ethanol concentration, the total caloric intake was standardized to 1000 kcal/kg, with a caloric distribution of 17% protein, 11% carbohydrate, 36% fat, and 36% ethanol. Lieber–DeCarli liquid diets were provided by Dooyeol Biotech (Seoul, Republic of Korea). For acute ethanol challenge, mice received a single oral gavage of ethanol (5 g/kg body weight) in the early morning and were sacrificed 9 h later. For probiotic administration, *L. helveticus* and *L. lactis* (CKD Bio, Seoul, Republic of Korea) and *V. dispar* (CKD Bio, Seoul, Republic of Korea) were suspended in phosphate-buffered saline (PBS) and administered twice weekly at a dose of 10^9^ CFU per mouse. At the end of the treatment period, mice were euthanized by isoflurane overdose (Aerane; Baxter, Deerfield, IL, USA). Body weight was recorded, and blood, liver, stool, and small intestine samples were collected. Whole blood (800 μL) was centrifuged at 19,000× *g* for 5 min to obtain serum. Liver and stool samples were rapidly excised and stored at −80 °C until analysis.

### 2.5. Endotoxin Assay

Mouse fecal samples were collected from 16-week-old control and treated mice 24 h after the last oral gavage. Fecal supernatants were prepared following a previously described protocol with minor modifications. Briefly, fecal samples were suspended in sterile PBS (1 g/10 mL for human; 50 mg/500 μL for mouse) and gently vortexed to minimize bacterial cell disruption. Suspensions were centrifuged at 3000 rpm for 15 min, and the supernatants were sequentially filtered through 0.45 μm and 0.22 μm filters. Samples were heat-inactivated at 90 °C for 15 min and stored at −80 °C until analysis. Endotoxin levels were determined using the Limulus Amebocyte Lysate (LAL) assay (Lonza, Basel, Switzerland, No. 50-647U) according to the manufacturer’s protocol. Plasma samples were diluted 10-fold, and fecal supernatants were diluted 10,000-fold in pyrogen-free water, followed by heat inactivation at 90 °C for 15 min. All measurements were performed in pyrogen-free glass tubes, Eppendorf tubes, or plates.

### 2.6. Real-Time PCR Analysis

Isolation of total RNA from tissue was performed using a Trizol reagent kit (Invitrogen, Gaithersburg, MD, USA) according to the manufacturer’s instructions. Aliquots of total RNA (2 μg) were converted to cDNA using the High-Capacity cDNA Reverse Transcription kit (Applied Biosystems, Foster City, CA, USA, Carlsbad, CA, USA). Real-time PCR was performed with Sybr Green using primer sets.

### 2.7. Quant-Seq Microarray

Total RNA quality was assessed using the Agilent 2100 Bioanalyzer with an RNA 6000 Nano Chip (Agilent Technologies, Amstelveen, The Netherlands), and RNA concentrations were measured with an ND-2000 spectrophotometer (Thermo Fisher Scientific, Waltham, MA, USA, Dover, DE, USA). Libraries were prepared using the QuantSeq 3′ mRNA-Seq Library Prep Kit (Lexogen, Vienna, Austria, Austria) according to the manufacturer’s instructions. Briefly, 500 ng of total RNA was hybridized with an oligo-dT primer carrying an Illumina-compatible sequence, followed by reverse transcription. After RNA template degradation, second-strand synthesis was performed using a random primer with an Illumina-compatible linker sequence. Double-stranded libraries were purified with magnetic beads and amplified to add complete the adapter sequences required for cluster generation. Final libraries were purified to remove PCR components. High-throughput sequencing was performed on an Illumina NextSeq 500 platform to produce single-end 75 bp reads. For data analysis, QuantSeq reads were aligned to either the genome assembly or representative transcript sequences using Bowtie2 (Langmead and Salzberg, 2012 [14]). Alignment files were used for transcript assembly, abundance estimation, and differential expression analysis. Differentially expressed genes (DEGs) were identified based on read counts from unique and multiple alignments using Bedtools (Quinlan, 2010 [15]). Read count data were normalized using the quantile normalization method implemented in edgeR (Bioconductor; Gentleman et al., 2004 [16]). Functional annotation of DEGs was performed using the Database for Annotation, Visualization, and Integrated Discovery (DAVID; https://davidbioinformatics.nih.gov/tools.jsp and Medline http://www.ncbi.nlm.nih.gov/, accessed date 14 November 2025). A porcine QuantSeq microarray was additionally performed using a customized service from eBiogen, Inc. (Seoul, Republic of Korea).

### 2.8. Circulating Bacterial DNA Extraction from Whole Blood

Add RBC lysis buffer to burst red blood cells from whole blood samples and incubate at 15 °C for 10 min. After centrifugation, remove the supernatant. When a white pellet is identified at the bottom of the tube, add 50 µL of 25 mM NAOH/0.2 mM EDTA buffer to dissolve it. Then boil the sample at 98 °C for 1 h to extract DNA from the cells. Cool this to 15 °C, then add 50 µL of 40 mM Tris-HCl (pH 5.5). Centrifuge the supernatant at 1500× *g* at 15 °C for 3 min. To quantify circulating bacteria, amplify DNA (1 μg) extracted from the blood using published primer sets for universal bacteria (forward: 5′-GTGSTGCAYGGYTGTCGTCA-3′; reverse: 5′-ACGTCRTCCMCACCTTCCTC-3′), *V. dispar* (forward: 5′-AACGCGTTGAAATTCGTCATGAAC-3′; reverse: 5′-GTGTAACAAGGGAGTACGGACC-3′) and Sybr green. Statistical analysis was performed with R version 3.6.3 on Windows 10 (http://www.R-project.org, accessed date 3 December 2025). The potential of *V. dispar* to distinguish patient groups was investigated using the relative abundance of *V. dispar* via q PCR in a receiver operating characteristic (ROC) curve analysis using p ROC package.3.

### 2.9. Statistical Analysis

Statistical analyses were performed using GraphPad Prism version 8.0.2 (GraphPad Software, San Diego, CA, USA). For continuous variables, group comparisons were conducted using one-way analysis of variance (ANOVA) followed by Sidak’s multiple comparison test. For high-dimensional data, such as microbiome taxonomy and RNA-seq gene expression, *p*-values were adjusted for multiple testing using the Benjamini–Hochberg False Discovery Rate (FDR) method. An FDR-adjusted *p*-value (q-value) < 0.05 was considered statistically significant. Data are expressed as mean ± SEM, and differences were considered statistically significant at *p* ≤ 0.05.

## 3. Results

### 3.1. Alterations to the Human Gut Microbiota in ALD

A total of 224 subjects were divided into four groups. Age, BMI, and blood biochemical parameters were recorded (Table 1). With the progression of ALD, serum AST, ALT, and γGTP levels increased. Triglycerides were elevated in AH patients compared with healthy controls, whereas creatinine, cholesterol, and HDL decreased in ALC patients (Table 2).

**Figure 1:**
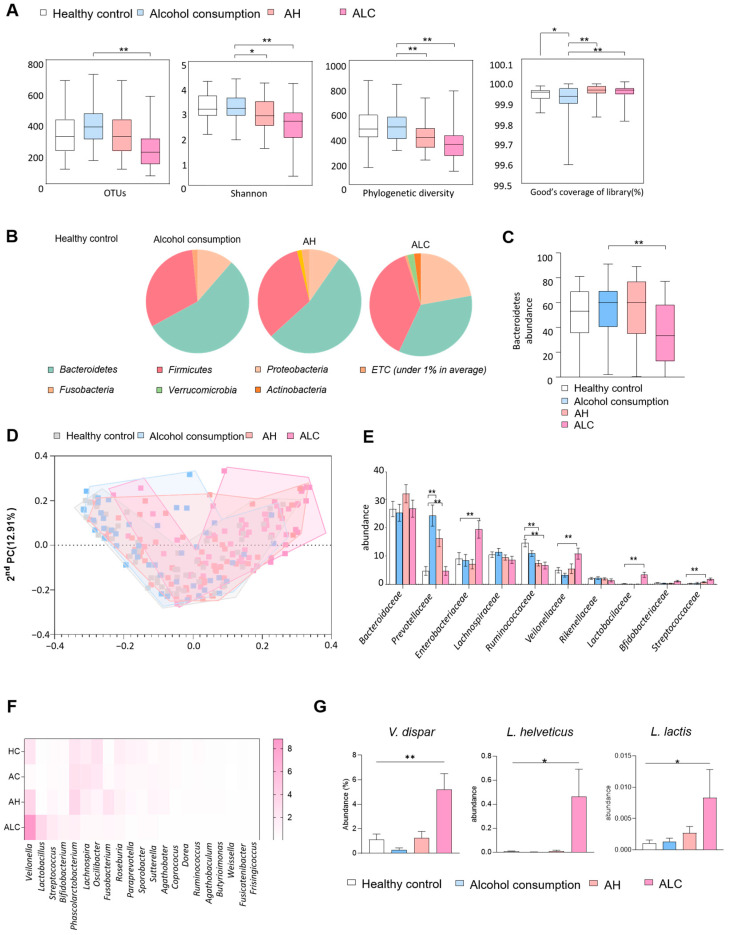
Gut microbiota was altered by alcohol consumption. Heathy control (*n* = 56), alcohol consumption (*n* = 46), AH (*n* = 57), and ALC (*n* = 65). Alpha diversity of (**A**) species richness estimator, including OTUs, ACE, CHAO, and Jackknife; (**B**) species diversity estimator (Shannon, NPShannon, Simpson, and phylogenetic diversity); (**C**) Good’s coverage of library and beta diversity with (**D**) PCoA plot showing dissimilarities in bacterial community structures based on Jensen–Shannon index. (**E**,**F**) Family and Genus composition. (**G**) *V.dispar*, *L. helveticus*, *L. delbreukii* (subsp. *L. lactics*) abundance. Statistical analysis was performed using a one-way ANOVA test (and nonparametric and mixed tests) and PCoA plots were generated using a nonparametric Kruskal–Wallis test. Data are given as median values (horizontal lines) with whiskers indicating 95% confidence intervals (Cis) (* *p* < 0.05, ** *p* < 0.01). AH, ALC.

We next analyzed gut microbiota composition across disease groups. Alpha-diversity indices, including observed OTUs, Shannon index, and phylogenetic diversity, progressively declined from healthy controls to ALC patients, while Good’s coverage was significantly higher in the ALC group (*p* < 0.001; Figure 1A).

At the phylum level, *Bacteroidetes* decreased and *Firmicutes* increased with disease severity, with a marked reduction in *Bacteroidetes* in ALC compared with healthy controls (*p* < 0.001; Figure 1B,C). Beta-diversity analysis by principal coordinate analysis revealed distinct clustering according to disease stage, with the ALC group showing the most pronounced separation (*p* < 0.001; Figure 1D). At the family level, ALC patients exhibited decreased *Ruminococcaceae* and *Prevotellaceae* but increased *Veillonellaceae*, *Lactobacillaceae*, *Enterobacteriaceae*, and *Streptococcaceae* compared with healthy controls (*p* < 0.01, *p* < 0.001; Figure 1E). Genus-level analysis further showed enrichment of *Veillonella* and *Lactobacillus* in the ALC group, as illustrated in the heatmap of dominant genera (Figure 1F). Notably, *V. dispar* displayed the greatest relative increase, while *L. helveticus* and the *L. delbrueckii* group *lactis* were also significantly elevated (*p* < 0.05, *p* < 0.01; Figure 1G). These findings suggest that *V. dispar* and specific *Lactobacillus* strains may serve as potential biomarkers or therapeutic candidates in ALD. To evaluate this hypothesis, we next investigated their effects in a mouse model of ALD.

### 3.2. L. helveticus and L. lactis Ameliorate Liver Injury and Intestinal Barrier Disruption in ALD

To evaluate the effects of *L. helveticus*, *L. lactis,* and *V. dispar* on alcohol-induced liver injury, candidate strains were orally administered to C57BL/6 mice fed a 5% ethanol-containing diet for 8 weeks. Gross and histological examination revealed severe steatosis and inflammation in the EtOH-fed group compared with controls. Treatment with *L. helveticus* and *L. lactis* improved liver morphology and reduced histological damage, whereas *V. dispar* had no effect. Histological scoring confirmed these results: the EtOH-fed group had the highest NAS (5.4), and *L. helveticus* and *L. lactis* significantly reduced NAS scores (2.6 and 3.1, respectively), approaching control levels (2.5).

**Figure 2:**
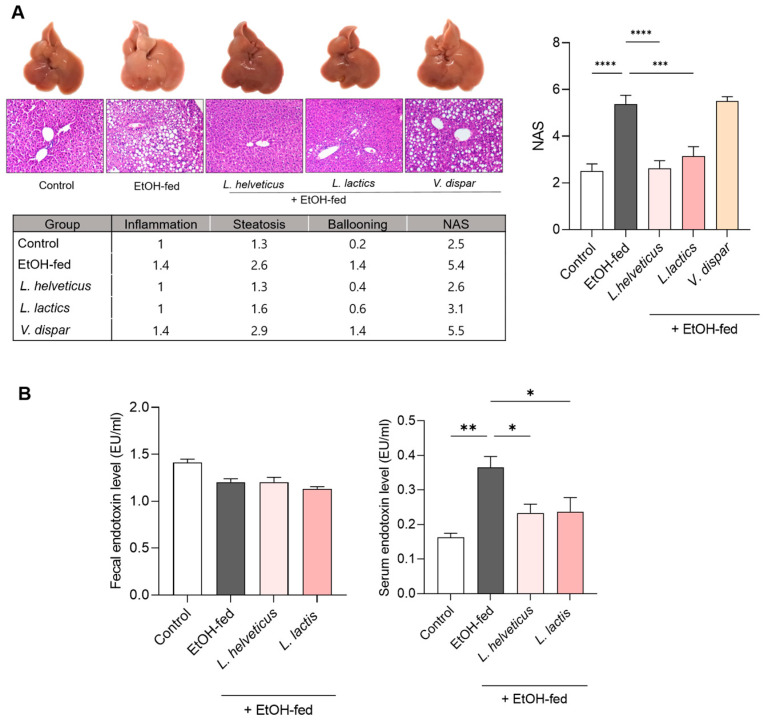
Effects of candidate bacterial strains on alcohol-induced liver injury. (**A**) Representative H&E-stained liver sections from control, EtOH-fed, *L. helveticus*, *L. lactis*, and *V. dispar* groups. Histological features include inflammation, steatosis, and ballooning degeneration. (**B**) Fecal and serum endotoxin level. Data are shown as the mean ± SEM. Statistical analysis was performed using a one-way ANOVA test (and nonparametric and mixed tests). (* *p* < 0.05, ** *p* < 0.01, *** *p* < 0.001, **** *p* < 0.0001).

In contrast, *V. dispar* failed to reduce injury, with an NAS of 5.5 comparable to EtOH-fed mice (Figure 2A). Serum AST, cholesterol, and triglyceride levels were also unchanged in the *V. dispar* group (Supplementary Figure S1). Based on these findings, *V. dispar* was excluded from further efficacy experiments. We next assessed whether *L. helveticus* and *L. lactis* improved gut barrier integrity and reduced endotoxemia. Fecal endotoxin levels did not differ among groups. However, serum endotoxin levels were significantly elevated in the EtOH-fed group compared with controls (*p* < 0.01) and were significantly reduced by *L. helveticus* and *L. lactis* treatment (*p* < 0.05; Figure 2B). Collectively, these findings indicate that *L. helveticus* and *L. lactis* ameliorated alcohol-induced liver injury by restoring intestinal barrier function. Guided by these results, we next examined the impact of these strains on gut microbiota composition.

### 3.3. L. helveticus and L. lactis Improve Gut Microbiota Composition and Function in ALD

**Figure 3:**
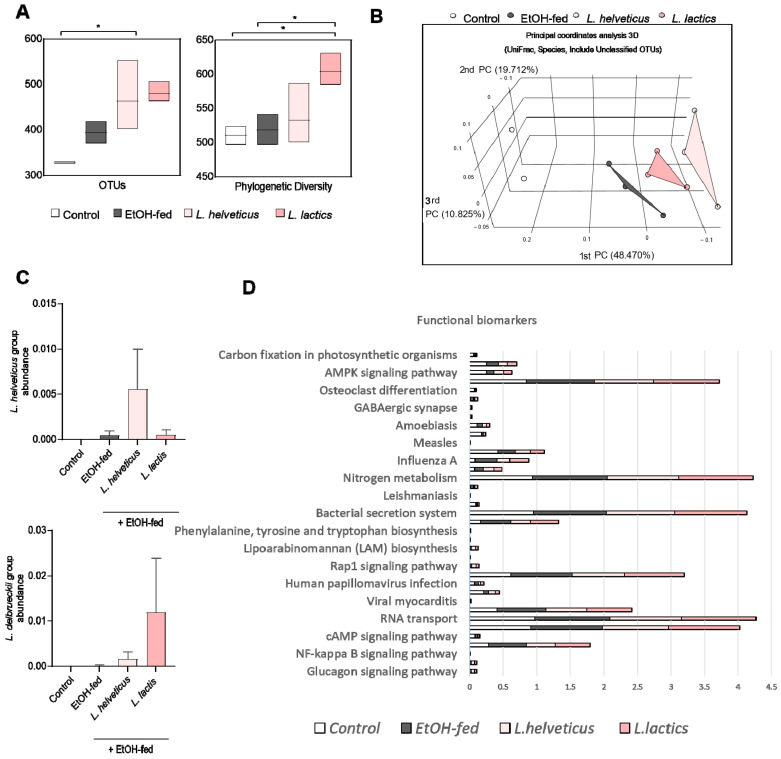
Candidate probiotics modulate gut microbiota in animal model. (**A**) Alpha-diversity was indicated using OTUs, ACE, Shannon, and phylogenetic diversity, and (**B**) beta-diversity was represented as a 3D PCoA plot showing the similarity of bacterial community structures based on UniFrac; 3D principal coordinate analysis (UniFrac and species, including unclassified OTUs). (**C**) Genus level of each group and the relative abundance of *L. helveticus* and *L. delbrueckii* (subsp. *L. lac*). (**D**) Alterations in gut microbiota-associated biofunctional markers following candidate probiotic treatment in an alcohol-induced liver disease model data are shown as the mean ± SEM. Statistical analysis was performed using a one-way ANOVA test (and nonparametric and mixed tests). (* *p* < 0.05).

We next evaluated the effects of *L. helveticus* and *L. lactis* on gut microbiota composition in alcoholic liver disease. Alpha-diversity, assessed by observed OTUs and phylogenetic diversity, was significantly reduced in the EtOH-fed group compared with controls but was restored to near-control levels by both *L. helveticus* and *L. lactis* treatment (*p* < 0.05; Figure 3A). Beta-diversity analysis using three-dimensional principal coordinate analysis (PCoA) revealed distinct clustering by treatment: the EtOH-fed group separated clearly from controls, whereas the *L. helveticus* and *L. lactis* groups formed clusters distinct from the EtOH-fed group (Figure 3B). At the genus level, EtOH-fed mice displayed a disrupted and heterogeneous microbial profile, whereas *L. helveticus* and *L. lactis* restored a more structured and balanced community. The abundances of *L. helveticus* and *L. lactis* were also significantly enriched in their respective treatment groups compared with controls and EtOH-fed mice (Figure 3C). Functional pathway analysis further demonstrated that the EtOH-fed group was enriched in pathways related to inflammation, infection, and metabolic disturbance, including NF-κB signaling, viral myocarditis, and bacterial secretion systems. Treatment with *L. helveticus* and *L. lactis* reduced these pathways and shifted the functional profile toward that of controls (Figure 3D). Collectively, these results suggest that *L. helveticus* and *L. lactis* not only modulate gut microbiota composition but also alleviate functional dysregulation induced by alcohol consumption.

### 3.4. L. helveticus and L. lactis Mitigate Hepatic Inflammation in ALD

**Figure 4:**
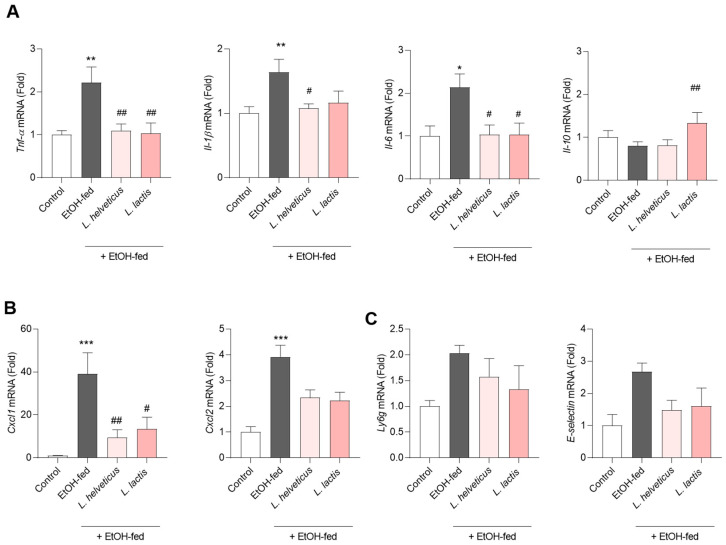
Probiotics suppress the inflammatory response caused by alcohol intake. (**A**) Probiotic groups Tnf-a, il-1b, il-6 and il-10 improved compared to the EtOH group. (**B**) Probiotic groups, chemokines and fibrosis-related genes. (**C**) ly6g and e-selectin subjected to real-time PCR. Values represent the mean ± SEM (*n* = 7–10). * *p* < 0.05, ** *p* < 0.01, and *** *p* < 0.001; # *p* < 0.05; ## *p* < 0.01; in comparison with the EtOH-fed group.

We next investigated the effects of *L. helveticus* and *L. lactis* on hepatic inflammatory gene expression in alcoholic liver disease. The EtOH-fed group showed significant upregulation of pro-inflammatory cytokines, including Tnf-α, Il-1β, and Il-6, compared with controls (*p* < 0.05–0.01). Treatment with *L. helveticus* and *L. lactis* significantly reduced these cytokines while restoring the expression of the anti-inflammatory cytokine Il-10, which was suppressed in EtOH-fed mice (Figure 4A). We also examined chemokines and fibrosis-related genes. Cxcl1 and Cox2 expression was markedly elevated in the EtOH-fed group but significantly decreased by probiotic treatment (Figure 4B). Markers of neutrophil adhesion and recruitment (Ly6g and E-selectin) showed no significant differences among groups, although expression tended to be lower in the *L. helveticus* and *L. lactis* groups compared with EtOH-fed mice (Figure 4C).

Collectively, these findings suggest that *L. helveticus* and *L. lactis* attenuate alcohol-induced liver inflammation.

### 3.5. L. helveticus and L. lactis Supplementation Mediates RNA Transcriptomic Profiles in the Liver

**Figure 5:**
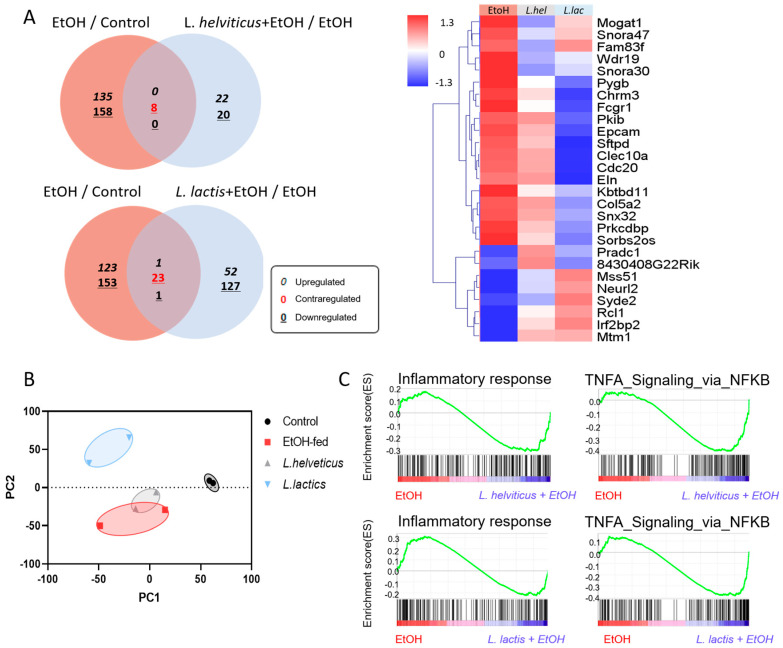
*L. hel* and *L. lac* supplementation mediates transcriptomic profiles in the liver. (**A**) Venn diagram showing overlapping DEGs among EtOH/control, *L. hel*/EtOH, EtOH/control and *L. lac*/EtOH, which were selected based on the cutoff value of fold change ≥ 2, normalized data (log2) ≥ 4.00, and *p*-value < 0.05 (*n* = 3 per group). (**B**) PCA plot of liver transcriptomes from control, EtOH-fed, *L. hel* and *L. lac* (*n* = 3 per group). Probiotic groups showed distinct clustering from the EtOH-fed group. (**C**) GSEA shows negative enrichment of inflammatory response and TNF-α signaling via NF-κB pathways in *L. hel-* and *L. lac*-treated groups compared to EtOH-fed mice.

To investigate the molecular mechanisms by which *L. helveticus* and *L. lactis* supplementation modulated liver inflammation, we performed RNA-seq on liver tissues from control, EtOH-fed, *L. helveticus* and *L. lactis* mice. Transcriptomic profiling revealed both overlapping and distinct differentially expressed genes (DEGs) among the groups, visualized by Venn diagrams and a heatmap (Figure 5A). Hierarchical clustering confirmed significant transcriptomic differences between probiotic-treated groups and the EtOH-fed group. Principal component analysis (PCA) further demonstrated clear separation among the four groups (Figure 5B). Gene set enrichment analysis (GSEA) identified significant pathway alterations in probiotic-treated groups, with inflammation-related pathways downregulated compared with the EtOH-fed group (Figure 5C). Together, these RNA-seq findings indicate that *L. helveticus* and *L. lactis* supplementation attenuates alcohol-induced hepatic inflammation through transcriptional reprogramming of inflammation-related pathways.

### 3.6. V. dispar Can Be a Marker for the Diagnosis of ALD

**Figure 6:**
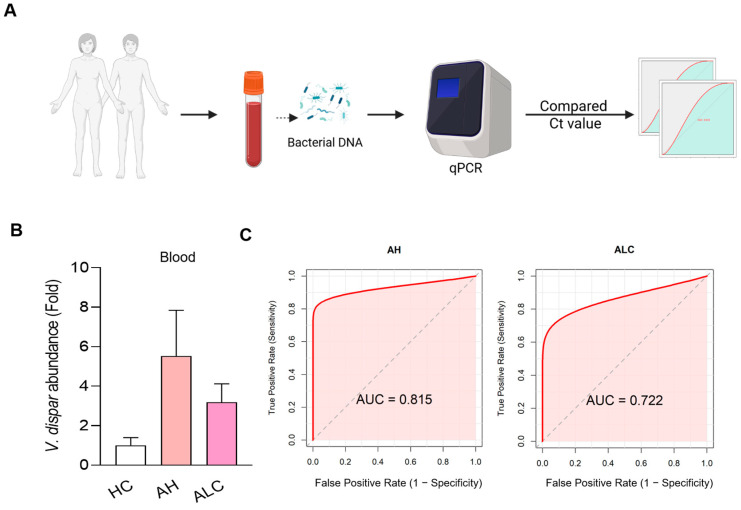
Diagnostic accuracy of *V. dispar* in human whole blood. (**A**) Schematic illustration of the workflow for quantifying *V. dispar* abundance in blood samples. Bacterial DNA was extracted from blood, and quantitative PCR (qPCR) was performed to compare Ct values among groups. (**B**) Relative abundance of *V. dispar* in blood from healthy control (HC), alcoholic hepatitis (AH), and alcoholic liver cirrhosis (ALC) patients. (**C**) AUROC curves for distinguishing AH from healthy controls (left) and AH + ALC from healthy controls (right) based on blood *V. dispar* abundance. The area under the curve (AUC) is indicated in each plot.

Based on the observation from Figure 1G that *V. dispar* was the most enriched genus in ALC patients, we hypothesized that circulating *V. dispar* levels could serve as a diagnostic marker for ALD. To test this, bacterial DNA was extracted from blood samples, and *V. dispar* abundance was quantified by qPCR in healthy controls (*n* = 15) and AH (*n* = 15) and ALC (*n* = 24) patients (Figure 6A) (Supplementary Table S1). The relative abundance of *V. dispar* was significantly higher in AH and ALC groups compared with healthy controls, with the highest levels detected in AH patients (Figure 6B). Receiver operating characteristic (ROC) analysis further demonstrated the diagnostic potential of *V. dispar*: the AUROC for distinguishing AH from controls was 0.815, and for ALC vs. controls it was 0.722, indicating moderate diagnostic accuracy (Figure 6C). These findings suggest that blood *V. dispar* quantification may provide a simple diagnostic tool for ALD, particularly for the early identification of patients with alcoholic hepatitis.

## 4. Discussion

We identified two major community-level alterations in the gut microbiota associated with the severity of ALD. First, α-diversity decreased, reflecting a loss of microbial richness and evenness, with overrepresentation of fewer taxa. Second, β-diversity was altered, indicating greater inter-individual variability in microbiome composition among patients with alcoholic liver cirrhosis. These changes were modest when comparing healthy controls, alcohol consumers, and AH patients but became more pronounced in comparisons between healthy controls and cirrhosis patients. Such increases in heterogeneity are consistent with previous reports in other microbiome-related disorders under various stress conditions [17,18]. Notably, our healthy controls were older than the patient groups. Despite the typical association of aging with inflammation, these older controls exhibited significantly better biochemical profiles (Table 2) than the younger AH and ALC groups. This conservative bias reinforces the fact that the observed alterations are driven primarily by disease pathology rather than age-related factors. Our findings suggest that the interplay between reduced species abundance, increased interpersonal diversity, and altered gut metagenome composition may play a critical role in ALD progression. Targeted regulation of heterogeneous microbial communities could therefore represent a potential strategy to mitigate disease development [19]. Contrary to other study, we found that the relative abundances of *Bacteroides* and *Prevotella* were not associated with disease progression [20,21]. Instead, *Veillonella* and *Lactobacillus* were enriched in patients with cirrhosis [18]. Previous studies have shown that translocation of the oral bacterium *Veillonella* contributes to gut microbiota alterations in cirrhosis patients [22]. Members of the *Veillonellaceae* family are lactate-utilizing bacteria that convert lactate into short-chain fatty acids (SCFAs) such as propionate [23], whereas *Lactobacillus* produces lactic acid and helps maintain specific microbial ecosystems. Chronic alcohol consumption is known to promote hepatic steatosis, which can progress to inflammation, fibrosis, and cirrhosis [24,25]. It is important to distinguish between the associative nature of our clinical findings and the causal evidence derived from our animal models. 

To evaluate potential protective effects, we administered *L. helveticus* and *L. lactis* to C57BL/6 mice exposed to ethanol. Eight weeks of alcohol feeding induced hepatic steatosis and inflammation, while probiotic supplementation modulated microbial community structures and mitigated alcohol-induced injury. Histological analysis confirmed that *L. helveticus* and *L. lactis* effectively attenuated liver inflammation associated with chronic alcohol exposure. Recent studies have confirmed that increased intestinal permeability promotes elevated serum LPS levels, and that this disruption of the gut barrier and subsequent release of inflammatory cytokines are closely associated with liver injury in ALD [26,27]. Consistent with these findings, our results confirmed that alcohol intake elevated circulating endotoxin, which in turn contributed to hepatic inflammation. Although not statistically significant, functional prediction analysis (Figure 3D) revealed a trend where ‘Bacterial secretion systems’ were enriched in the EtOH-fed group but reduced by probiotic treatment. This pattern biologically aligns with our phenotypic findings, suggesting that *L. helveticus* and *L. lactis* may mitigate hepatic inflammation by dampening bacterial virulence and subsequent NF-κB signaling. To elucidate how these strains, differ mechanistically from other probiotics, we further analyzed hepatic transcriptomic profiles. While standard probiotics often function by modulating the gut microbiota composition, our RNA-seq data indicate that *L. helveticus* and *L. lactis* exert a direct immunomodulatory effect on the liver. Specifically, these strains induced a transcriptional reprogramming of hepatic immune cells. We observed the upregulation of *Fcgr1*, *Clec10a*, and *Irf2bp2*. The current literature identifies *Clec10a* as a specific marker of M2 macrophages, which are crucial for tissue repair and immune tolerance [28]. Furthermore, *Irf2bp2* acts as a transcriptional co-repressor that limits excessive inflammatory responses by inhibiting NF-κB signaling [29]. This suggests that *L. helveticus* and *L. lactis* do not merely reduce the pathogenic burden from the gut but actively condition hepatic Kupffer cells to adopt a tolerogenic phenotype. Downregulation of pro-inflammatory mediators and restoration of genes linked to epithelial integrity and immune tolerance suggest that *Lactobacillus* strains may reduce hepatic inflammation by attenuating the activation of Kupffer cells and neutrophil recruitment [30]. Collectively, these transcriptomic alterations point toward a rebalancing of the hepatic immune microenvironment, supporting a shift from a pro-inflammatory state induced by ethanol toward a more homeostatic and anti-inflammatory profile under probiotic intervention [21]. In our study, alcohol intake synergistically promoted chronic inflammation through upregulation of Cxcl1 mRNA-enhanced hepatic immune function. Notably, treatment with the candidate probiotic strains inhibited these signaling events, suggesting their potential to prevent liver damage associated with chronic alcohol consumption.

Previous studies have shown that *Veillonella* is enriched in the gut microbiome of elite athletes and has been associated with performance-enhancing activity [31]. In contrast, Chen et al. and Oh et al. reported enrichment of *Veillonella* among cirrhotic patients. This demonstrates that the functional role of *Veillonella* in liver disease has not been clearly established [32]. In the present study, we evaluated the function and potential of *V. dispar,* a gut-resident bacterium detectable in circulation. No evidence of disease exacerbation was observed following *V. dispar* administration in our experimental models (Supplementary Figure S1).

While *Veillonella* is a common oral commensal [22] and could theoretically enter the circulation from the oral cavity, activity in the gut and blood strongly suggests that the gut reservoir is the major source of circulating *V. dispar* in these patients. Indeed, bacterial biomarkers derived from the gut microbiome have already been investigated in relation to hepatic steatosis and fibrosis [33,34]. Although we excluded patients with recent antibiotic use and major comorbidities, we could not strictly control for individual dietary factors or the use of artificial sweeteners, which may influence the microbiome. However, the consistent enrichment of *V. dispar* across the disease spectrum suggests that ALD pathology exerts a stronger influence on the gut ecosystem than these external factors. Although our study has limitations, the diagnostic accuracy (AUROC) of blood *V. dispar* for distinguishing alcoholic hepatitis and cirrhosis was 0.815 and 0.72, respectively. While results were not fully consistent between fecal and blood samples, these findings suggest that *V. dispar* has potential as a biomarker for screening and monitoring in alcoholic liver disease. Traditional biochemical markers such as AST, ALT, and GGT are widely used for screening ALD, but they primarily reflect hepatocellular damage and can be influenced by various extrahepatic factors. In our study, *V. dispar* achieved an AUC of 0.815 for distinguishing alcoholic hepatitis (AH) from healthy controls, which indicates good diagnostic accuracy comparable to these classical markers. Further studies are warranted to validate its diagnostic utility.

## 5. Conclusions

In conclusion, our study highlights two clinically actionable targets for ALD: *V. dispar* as a non-invasive diagnostic biomarker, and *L. hel* and *L. lac* as therapeutic agents to attenuate hepatic inflammation.

## Figures and Tables

**Table 1:** Flow diagram of enrolled patients. ALD, alcoholic liver disease; AH, alcoholic hepatitis; ALC, alcoholic liver cirrhosis.

Classification	Groups	*n*
Controls	Healthy controls	56
	Alcohol consumption	46
ALD Patients	AH Patients	57
	ALC Patients	65

**Table 2:** This table compares the clinical characteristics and biochemical parameters among healthy controls, alcohol consumers, and patients with alcoholic hepatitis (AH) and alcoholic liver cirrhosis (ALC). (* *p* < 0.05, ** *p* < 0.01, *** *p* < 0.001).

	Healthy Control (*n* = 56)	Alcohol Consumption (*n* = 46)	AH (*n* = 57)	ALC (*n* = 65)
Age (years)	61.59 (±1.26)	58.72 (±0.89)	52.20 *** (±1.78)	54.30 *** (±1.29)
BMI (kg/m^2^)	23.00 (±0.50)	23.94 (±0.42)	24.13 (±0.59)	23.74 (±0.43)
AST (U/L)	22.40 (±0.66)	23.65 (±0.91)	82.49 ** (±13.49)	116.6 *** (±21.11)
ALT (U/L)	18.33 (±1.00)	20.85 (±1.31)	63.91 *** (±6.50)	44.37 ** (±1.26)
Creatine (mg/dL)	0.92 (±0.30)	0.85 (±0.03)	0.91 (±0.02)	0.79 ** (±0.03)
Cholesterol (mg/dL)	175.3 (±5.32)	193.7 (±5.22)	166.2 (±6.80)	131.2 *** (±6.40)
γGTP (IU/L)	40.40 (±14.87)	47.35 (±7.04)	299.9 * (±60.72)	444.6 *** (±103.7)
Triglyceride (mg/dL)	117.2 (±14.96)	145.0 (±12.09)	234.6 *** (±28.39)	141.5 (±16.10)
HDL (mg/dL)	54.56 (±2.63)	58.00 (±2.54)	54.77 (±3.15)	34.40 *** (±2.96)

## Data Availability

The data presented in this study are available upon request from the corresponding authors. The data are not publicly available due to ethical and privacy reasons.

## Supplementary Materials

The following supporting information can be downloaded at https://www.mdpi.com/article/10.3390/microorganisms14020449/s1, Figure S1: *V. dispar* does not have the effect on alcohol liver disease; Figure S2: Graphical abstract; Table S1: Patients characteristics for diagnostic marker.

## Abbreviations

The following abbreviations are used in this manuscript:

ALD	alcohol-associated liver disease
CXCL1	chemokine (C-X-C motif) ligand 1
*V.dispar*	*Veillonella dispar*
PAMPs	pathogen-associated molecular patterns
Abx	antibiotics
*L. helveticus*	*Lactobacillus helveticus*
*L. lactics*	*Lactobacillus lactics*
16S rDNA	16S ribosomal DNA
AST	aspartate transaminase
ALT	alanine transaminase
TG	triglyceride
γGTP	gamma-glutamyl transpeptidase
AUC	area under the ROC curve
PCoA	principal coordinate analysis
