# Supplementary material for: Microbial Biomarkers for the Prevention and Diagnosis of Alcoholic Liver Disease
Source: Microorganisms. 2026 Feb 12;14(2):449. doi: 10.3390/microorganisms14020449 (PMC12942687; doi:10.3390/microorganisms14020449)
Supplement: Supplementary file 1 [file microorganisms-14-00449-s001.zip › microorganisms-4122507-supplementary.pdf]

Supplementary file

Variables (mean [SEM])	AH (*n=15*)		ALC (*n=24*)	
BMI (kg/m^2)	24.75	(0.99)	24.46	(0.65)
AST (U/L)	122.1	(28.49)	96.13	(12.40)
ALT (U/L)	100.1	(23.41)	24.94	(2.92)
Creatine (mg/dL)	0.72	(0.03)	0.86	(0.07)
Cholesterol (mg/dL)	195.8	(12.40)	145.5	(10.36)
γGTP (IU/L)	407.4	(112.2)	416.2	(97.49)
Triglyceride (mg/dL)	448.2	(177.6)	141.0	(27.69)
HDL (mg/dL)	50.45	(5.83)	70.00	(5.32)
LDL (mg/dL)	89.45	(11.90)	65.25	(22.17)

Supplementary Table S1. Patients characteristics for diagnostic marker.

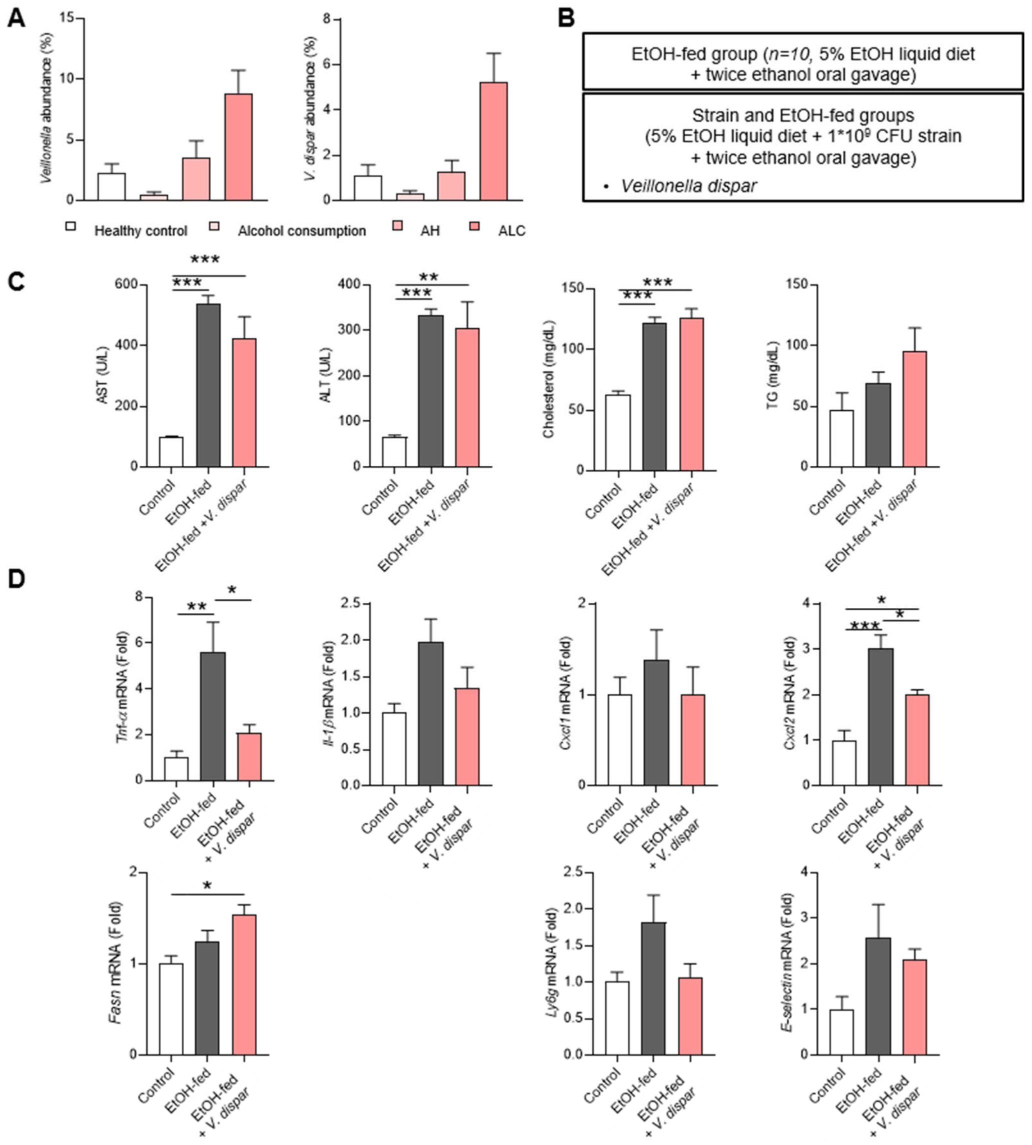

Supplementary Figure S1. *V. dispar* does not have the effect on alcohol liver disease. A. Relative abundance. B. Animal experiment. C. Serum levels of AST, ALT (U/L), total cholesterol, and triglycerides (TG; mg/dL) were measured in three groups: Control, EtOH-fed, and EtOH-fed + V. dispar. D. Hepatic mRNA expression of inflammatory/chemokine genes (Tnf-α, Il-1β, Cxcl1, Ccl2) and metabolism/stress and endothelial activation–related genes (Fasn, Ucp2, E-selectin) was quantified by qRT–PCR and presented as fold change relative to Control. Bars represent mean ± SEM. Statistical significance was determined by one-way ANOVA followed by a post hoc multiple-comparison test (e.g., Tukey). P<0.05, P<0.01, P<0.001, P<0.0001.

Data are shown as the mean ± SEM. Statistical analysis was performed using a One-Way ANOVA test (and Nonparametric or Mixed). (**P < 0.01, ***P < 0.001)

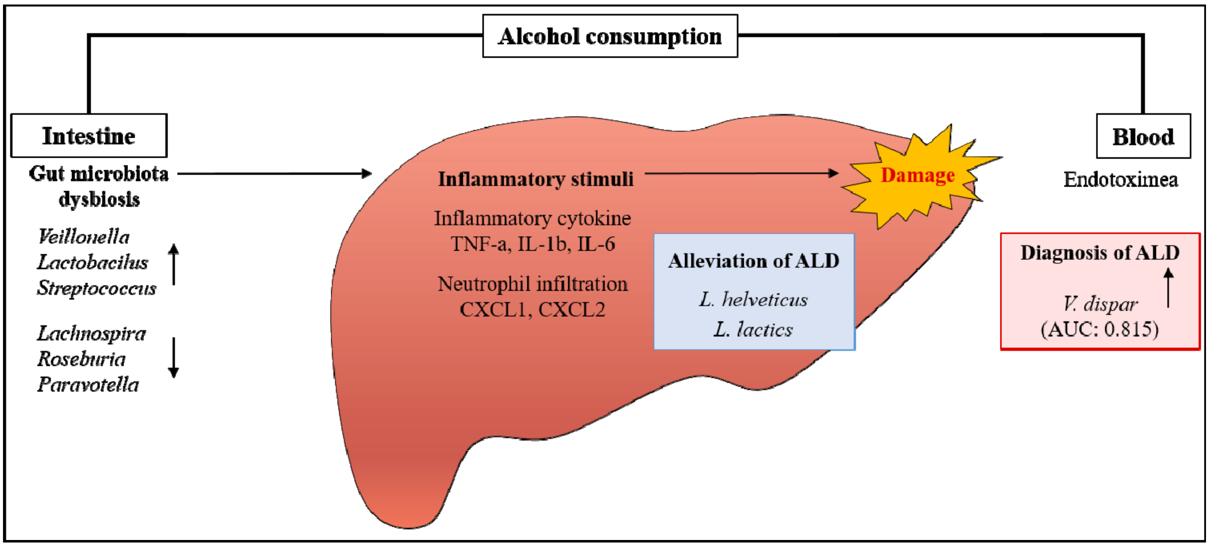

Supplementary Figure S2. Graphical abstract
